# Development and Validation of Serotype-Specific Blocking ELISA for the Detection of Anti-FMDV O/A/Asia1/SAT2 Antibodies

**DOI:** 10.3390/v16091438

**Published:** 2024-09-10

**Authors:** Mohammad A. Kashem, Patrycja Sroga, Vivien Salazar, Hamza Amjad, Kate Hole, Janice Koziuk, Ming Yang, Charles Nfon, Shawn Babiuk

**Affiliations:** National Centre for Foreign Animal Disease, Canadian Food Inspection Agency, Winnipeg, MB R3E 3M4, Canada; mohammad.kashem@inspection.gc.ca (M.A.K.); patrycja.sroga@inspection.gc.ca (P.S.); vivien.salazar@inspection.gc.ca (V.S.); hamza.amjad@inspection.gc.ca (H.A.); kate.hole@inspection.gc.ca (K.H.); janice.koziuk@inspection.gc.ca (J.K.); ming.yang@inspection.gc.ca (M.Y.); charles.nfon@inspection.gc.ca (C.N.)

**Keywords:** FMDV serotypes, monoclonal antibody, polyclonal antibodies, bELISA, DSp, DSe

## Abstract

Foot-and-mouth disease (FMD) is one of the most infectious viral transboundary diseases of livestock, which causes devastating global economic losses. Different enzyme-linked immunosorbent assays (ELISAs) are used for sero-surveillance of the foot-and-mouth disease virus (FMDV). However, more sensitive, accurate, and convenient ELISAs are still required to detect antibodies against FMDV serotypes. The primary goal of this study was to establish serotype-specific monoclonal antibody (mAb)-based blocking ELISAs (mAb-bELISAs) that would provide better performance characteristics or be equivalent in performance characteristics compared with a conventional polyclonal antibody (pAb)-based competitive ELISA (pAb-cELISA). Four mAb-bELISAs were developed using FMDV serotype-specific mAbs for the detection of anti-FMDV/O/A/Asia1/SAT2 antibodies. Using a 50% cut-off, all four mAb-bELISAs exhibited species-independent 99.74%, 98.01%, 96.59%, and 98.55% diagnostic specificity (DSp) and 98.93%, 98.25%, 100%, and 87.50% diagnostic sensitivity (DSe) for FMDV serotypes O, A, Asia1, and SAT2, respectively. In addition, a 100% DSe of serotypes O- and SAT2-specific mAb-bELISAs was observed for porcine sera when the cut-off was 30%. All mAb-bELISAs developed in this study displayed high repeatability/reproducibility without cross-reactivity. Finally, the diagnostic performance of mAb-bELISAs was found to be better than or equivalent to compared with pAb-cELISAs, suggesting that mAb-bELISAs can be used to replace existing pAb-ELISAs for the detection of antibodies against these four FMDV serotypes.

## 1. Introduction

Foot-and-mouth disease (FMD) is a highly transmissible viral disease that affects mostly cloven-hoofed animals of different species, such as pigs, cattle, sheep, goats, buffalo, and deer [1]. It causes devastating economic losses due to trade restrictions and recovery efforts from outbreaks [2,3]. The foot-and-mouth disease virus (FMDV) is the causal agent of FMD, which is classified under the family *Picornaviridae* and genus *Aphthovirus* [4]. FMDV is a non-enveloped positive-sense single-stranded RNA (ssRNA) virus, whose genome consists of 12 proteins, namely 4 structural (VP1-4) and 8 non-structural proteins (L, 2A–C, and 3A–D) [5]. To date, seven genetically and antigenically distinct FMDV serotypes, O, A, C, Asia1, South African Territory (SAT)1, SAT2, and SAT3, have been identified [6,7]. FMDV serotypes O and A are present in most of the FMDV pools except for pool 6, Southern Africa. Serotype C is assumed to be extinct globally since the last detection in 2004 [8,9,10,11]. Serotypes Asia1 and SAT1-3 circulate mainly in Asia and Africa, respectively [8]. The control of FMD primarily depends on the vaccination of susceptible animals with inactivated FMD whole-virus vaccines together with movement restrictions of infected animals [12,13,14,15,16]. 

The enzyme-linked immunosorbent assay (ELISA) has been a preferred method for the serodiagnosis of different FMDV serotypes due to its efficiency, diagnostic specificity, and sensitivity [17,18,19]. Various formats of ELISAs have been used to identify FMDV serotypes, including blocking ELISA (bELISA) [20,21], competitive ELISA (cELISA) [22], and double-antibody sandwich ELISA (DAS-ELISA) [23]. Serotype-specific rabbit or guineapig polyclonal antibodies (pAbs) or antiserum used as both capture and detection antibodies are the most common tools in ELISAs for FMDV serotypes [24]. However, due to the presence of many complex components in pAbs or antisera, their use in ELISA is not ideal due to unwanted cross-reactivity. In addition, pAbs are hard to produce on a large scale and hard to standardize due to their heterogeneity, resulting in limited large-scale application [1]. Thus, alternatives to pAbs, for example, monoclonal antibodies (mAbs), are a promising approach to making reproducible and convenient ELISAs for serological diagnostics of FMDV serotypes.

Laboratory-produced mAbs are valuable reagents in research, disease diagnosis, and therapeutic interventions [25]. Highly specific mAbs used in diagnostic ELISAs can improve the overall accuracy, specificity, and sensitivity in comparison with pAb-based ELISAs [25]. The application of mAbs further maintains consistent assay quality owing to their large-scale availability and minimal batch-to-batch variability [25]. In this study, FMDV serotype-specific mAb-based blocking ELISAs (mAb-bELISAs) were developed using in-house developed mAbs compared with the conventional pAb-based competitive ELISA (pAb-cELISA). Four mAbs were identified as suitable to detect serotype-specific antibodies against FMDV in bELISAs for O, A, Asia1, and SAT2. In the mAb-bELISA, serotype-specific antibodies, if present in animal sera, bind to the specific FMDV antigens coated on the ELISA plate, which subsequently inhibits the binding of HRP (horseradish peroxidase)-conjugated mAbs at the same epitopes/regions, resulting in reduced optical density (OD) values for positive sera. The diagnostic specificity (DSp) of the mAb-bELISAs was calculated by testing negative animal sera from Canada and the diagnostic sensitivity (DSe) was estimated by testing sera from experimentally infected, contact-exposed, and/or vaccinated-challenged animals.

## 2. Materials and Methods

### 2.1. Ethics Statement 

All animal studies, including animal use and study procedures (animal use document C-02-002), were approved by the Canadian Science Centre for Human and Animal Health Animal Care Committee (CSCHAH-ACC). All study procedures, including animal inoculation and care, were conducted according to the appropriate guidelines of the Canadian Council of Animal Care (CCAC).

### 2.2. Serum Samples

Negative sera were collected from Canadian porcine (n = 867), bovine (n = 555), ovine (n = 608), and caprine (n = 593) species. Positive porcine (n = 105), bovine (n = 93), and ovine (n = 119) sera were collected from experimentally infected, contact-exposed, or vaccination-challenged animals at different days post-inoculation (dpi) or days post-vaccination (dpv). All positive sera were raised against FMD O/A/Asia1/SAT2 serotypes, including the following strains: O/UKG/11/2001, O/Manisa/TUR/69, A24/Cruzeiro/Br/55, A22/IRQ/24/64, Asia1/Shamir, SAT2/SAU 1/2000, and SAT2/ZIM/5/81, at the National Centre for Foreign Animal Diseases, Canadian Food Inspection Agency (NCFAD/CFIA). Inoculation of FMDV strains into specific animals (cattle, sheep, and pigs) was performed as previously described [12,26]. In brief, the experimentally infected animal study included an infected animal group and a control group. Sera were typically collected at −1 dpi or 0 dpi (also named pre-inoculation/challenge collection), followed by regular post-inoculation collections (usually every day or every other day). Typical endpoints for this study were 21–28 dpi. Experiments involving contact-exposed animals were typically conducted by initially separating each animal group before the challenge, and then moving the contact-exposed animals into the cubicle housing with the challenged animals. Sera were collected similarly to the experimentally infected animal studies. In the case of the vaccine efficacy testing experiments, a test group (vaccinated and then challenged) and a control group (no vaccine, only challenged) were employed. Monovalent FMDV serotype-specific inactivated vaccines were mostly used to vaccinate animals in this study. Following vaccination, serum samples were collected typically at 0-, 7-, 14-, 21-, and 28-dpv. Both vaccinated and control animals were then challenged/inoculated with viruses and sera collections took place multiple times, including daily, every other day, or weekly. 

### 2.3. Production of Monoclonal Antibodies (mAbs)

FMDV serotype-specific mAbs were produced in-house by using hybridoma technology, as previously described [25,27,28]

### 2.4. Conjugation of FMDV Serotype-Specific mAbs with HRP 

Mouse anti-FMDV serotype-specific mAbs were conjugated with HRP (horseradish peroxidase) using a Lightning-Link^®^ kit according to the manufacturer’s instructions (Abcam, cat# ab102890). Briefly, all reagents were equilibrated at room temperature for 10–15 min and agitated before use. First, 1 µL of modifier reagent was added to 10 µL of mAb (1 mg/mL), mixed gently by inversion, and then directly added to the lyophilized HRP mix. The mixture was resuspended by pipetting up and down two times and incubated at room temperature (RT; dark room) for at least 3 h or overnight. Following incubation, 1 µL of quencher reagent was added to the antibody-HRP mixtures, mixed gently by inversion, and left in a dark room at RT for 30 min. Afterward, the conjugated antibody was used immediately or stored at 4 °C for 18 months or long-term storage with 50% glycerol at −20 °C.

### 2.5. Development of FMDV Serotype-Specific mAb-bELISAs

Serotype-specific mAb-based blocking ELISAs (bELISAs) for FMDV were developed as described earlier, with a few modifications [29]. 

In brief, for FMDV serotype O mAb-bELISA, a 96-well Maxisorp ELISA plate was coated with 100 µL/well of rabbit anti-FMDV O1/Manisa polyclonal antibody (1:10,000), used as a capture antibody, diluted homogenously in coating buffer (0.06 M carbonate buffer, pH 9.6 ± 0.1). The plate was sealed with an adhesive plate sealer and incubated at 4 °C overnight. After 5× washes with washing buffer (PBS-T: 0.01 M PBS [phosphate-buffered saline] plus 0.05% Tween 20, pH 7.2 ± 0.1), 100 µL of inactivated FMDV antigens (O1/Manisa [1:175] diluted in 1× casein blocking buffer (Sigma Aldrich, St. Louis, MO, USA; cat#B6429) was added to each well of the plate, sealed with a plate sealer, and incubated at 37 °C for 1 h with gentle shaking (~60 rpm). Following 5× washes, 50 µL of test sera (1:5 dilution) and control sera (Q1 [C138 O1/Manisa, Q2 [1:4 C138 O1/Manisa,), Q3 [normal bovine serum-undiluted], and DC [dilution control]) diluted in blocking buffer were added to the designated wells and incubated at 37 °C for 1 h with gentle shaking. After another 5× washes, serotype-specific HRP-conjugated purified mouse anti-FMDV mAbs (F21-FMDO-64-2-1 [1:2000]) diluted in blocking buffer were added to each well and incubated at 37 °C for 1 h with gentle shaking. Following a final 5× wash, 100 µL of TMB substrates (equal volume of substrate A and B; SeraCare, Milford, MA, USA; cat# 5120-0038 and 5120-0049) were added to each well and incubated at RT (in the dark) for 10 min with gentle shaking. Immediately after incubation, the reaction was stopped by adding 100 µL of TMB stop solution (SeraCare, Milford, MA, USA; cat# 5150-0021), and the plate was read by SpectraMax PLUS (Molecular Devices, San Jose, CA, USA). The OD value was measured at 450 nm and the PI (percentage of inhibition) for each sample was calculated by using the following formula:

PI = (1 − [test sample OD/diluent control OD]) × 100%.

A set target for ODs and PIs of positive and negative controls was used in each ELISA plate. OD value ranges from 0.9 to 3.0 were considered for DC and Q3 (negative) controls and PI values of 50% (at least) and 90% were considered for Q2 and Q1 controls, respectively.

The same protocol described for FMDV/O above was used for the development of FMDV A, Asia1, and SAT2 using corresponding serotype-specific reagents at optimal dilutions, as shown in Table 1 below.

### 2.6. Competitive ELISAs with mAbs (mAbs-cELISA) and pAbs (pAb-cELISA)

Competitive ELISAs using mAbs were performed similarly to the procedures applied for mAb-bELISA except for the incubation of test and control sera with mouse anti-FMDV mAbs (F21-FMDO-64-2-1, F66-A22-14, F34-Asia1, or F76-SAT2-11-2-1) at the same time, followed by the addition of goat anti-mouse IgG-HRP (1:2500 dilution; Jackson ImmunoResearch, West Grove, PA, USA; Cat# 115-035-146) for 1 h at 37 °C. Similarly, competitive ELISAs using pAb were conducted by incubating test and control sera with guineapig anti-FMD Ab (1:10,000 dilution, in-house developed) followed by incubation with donkey anti-guineapig IgG-HRP (1:2500 dilution; Jackson ImmunoResearch, West Grove, PA, USA; Cat# 706-035-148) for 1 h at 37 °C and then with SIGMAFAST OPD (o-Phenylenediamine) substrate (Sigma-Aldrich, St. Louis, MO, USA; Cat# P9187). The OPD substrate reaction was stopped by adding 100 µL of 2 M sulfuric acid.

### 2.7. Diagnostic Specificity (DSp) and Sensitivity (DSe) of the mAb-bELISAs

The guidelines from the World Organisation for Animal Health (WOAH) *Terrestrial Manual* (2022) were followed to validate the mAb-bELISAs. For all FMDV serotype-specific mAb-bELISAs, we aimed to achieve at least 95% DSp and 95% DSe with a confidence of 95% (5% error allowed). The DSp of the assay was calculated by testing known negative serum samples (n = 760 for serotype O; n = 1108 for serotype A; n = 938 for serotype Asia1; and n = 1031 for serotype SAT2) from different animal species (porcine, bovine, ovine, and caprine), and the DSe was estimated by testing serum samples from experimentally infected or vaccinated animals (n = 99 for serotype O; n = 114 for serotype A; n = 33 for serotype Asia1; and n = 56 for serotype SAT2). The optimal assay cut-off, which provides relatively high DSp and DSe, was determined from the PI distribution according to the procedure described elsewhere [30]. The cut-off value for all four assays was calculated as follows: mean PI of negative sera+ (3× standard deviations). An arbitrary 50% cut-off was also considered when PI values were exceptionally high for known negative sera. The optimal DSp and DSe of each serotype-specific assay were calculated based on the set PI cut-off values.

### 2.8. Virus Neutralization Test (VNT)

False positives in known negative sera (above the set cut-off) and false negatives in known positive sera (below the set cut-off) identified in mAb-bELISAs specific to FMDV serotypes O, A, Asia1, and SAT2 were confirmed by VNT on IB-RS-2 monolayer cells (a porcine kidney cell line) using known FMD viruses as described elsewhere [31]. In brief, serial two-fold dilution test sera were incubated with 100 TCID_50_ (tissue culture infectious dose 50) FMD viruses (serotype-specific) in a 96-well sterile tissue culture plate and incubated at 37 °C with 5% CO_2_ for 1 h. Immediately following incubation, 5 × 10^4^ cells/well of IB-RS-2 cells were added to each well and incubated at 37 °C with 5% CO_2_ for 2–3 days. The CPE (cytopathic effect) was examined under the microscope and recorded. The endpoint titer of neutralizing antibodies was calculated as the reciprocal of the last serum dilution to neutralize 100 TCID_50_ FMD viruses in 50% of the wells.

### 2.9. Repeatability/Reproducibility of mAb-bELISAs

To confirm the stability of FMDV serotype-specific mAb-based bELISAs, weak positive control sera (Q2) used for each serotype were examined in duplicate in the same microplate (intra-assay) and microplates in different production batches (inter-assay) under optical parameters. The percentage coefficients of variation (%CV) of replicate sera were calculated; <10% and <15% CVs were maintained for intra-assay and inter-assay repeatability/reproducibility, respectively. 

### 2.10. Data Analysis

Raw OD values from each plate were organized and subsequently converted to PI values using Microsoft Office Excel 2016. Normalization of PI values, generation of scatter plots, and frequency (n) distribution (bar graph) for estimating cut-off line (target DSp and DSe of minimum 95% with 5% CI [confidence interval]) were also carried out in Microsoft Office Excel 2016. Receiver operating characteristic (ROC) curve and bar graph analysis were conducted in GraphPad Prism Version 10.2.2. In the ROC curve, the area under the curve (AUC) was 1.0. When the ROC curve completely hugged the top left corner of the plot, the AUC equaled 1.0, indicating a high accuracy of the assay in classifying positive and negative sera that provide optimal DSe and DSp. 

## 3. Results

### 3.1. Diagnostic Specificity (DSp) and Sensitivity (DSe) of FMDV/O mAb-bELISA

**Figure 1:**
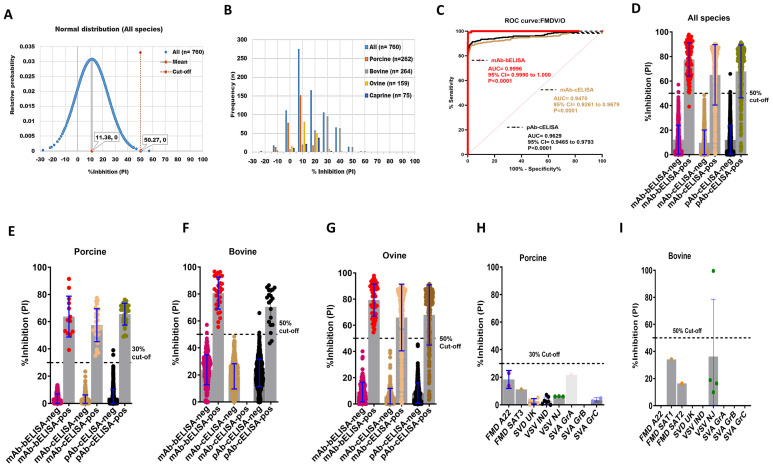
DSp and DSe of FMDV/O-specific mAb-bELISA and comparator ELISAs. (**A**) Normal distribution of PI obtained from negative serum samples of Canadian healthy animal species (n = 760; porcine, bovine, ovine, and caprine). (**B**) Frequency distribution of PI in negative animal sera (n = 760 [all species], n = 262 [porcine], n = 264 [bovine], n = 159 [ovine], and n = 75 [caprine]). (**C**) ROC curve diagnostic sensitivity analysis of FMDV/O mAb-bELISA in contrast to mAb-cELISA and pAb-cELISA. (**D**–**G**) Bar graphs show the estimated PI cut-off line and overall DSe of FMDV/O mAb-bELISA and comparator ELISAs for all animal species (**D**), porcine (**E**), bovine (**F**), and ovine (**G**). (**H**,**I**) Cross-reactivity analysis of FMDV/O mAb-bELISA using sera from experimentally infected animals, such as porcine (**H**) and bovine (**I**), with other FMDV serotypes and other vesicular diseases. In (**A**), the red vertical dashed line and grey vertical solid line represent estimated PI cut-off and mean values, respectively. Dashed lines in all bar graphs indicate the estimated PI cut-off.

To assess the DSp of FMDV/O mAb-bELISA, 760 known negative sera collected from naive porcine (n = 262), bovine (n = 264), ovine (n = 159), and caprine (n = 75) were used. The results showed a normal distribution for PI from all negative sera and the average cut-off was at 50.27%, ranging from 19.67% (porcine) to 59.78% (bovine) (Figure 1A,B and Figure S1A–D). Therefore, a 50% cut-off was considered for optimal DSp, which provided an overall 99.74% DSp of the assay (Table 2). In particular, mAb-bELISA scored 100%, 99.24%, 100%, and 100% DSp in porcine, bovine, ovine, and caprine sera, respectively (Table 2). 

The DSe of FMDV/O mAb-bELISA was determined using sera from FMDV/O experimentally infected, contact-exposure, and vaccinated-challenged animals (porcine, bovine, and ovine) collected at different time points (0–30 dpi). The ROC curve analysis revealed an optimal DSe of FMDV/O mAb-bELISA (AUC = 0.9996; std. error = 0.0003012; 95% CI = 0.9990 to 1.000; *p* < 0.0001) compared with mAb-cELISA (AUC = 0.9470; std. error = 0.01069; 95% CI = 0.9261 to 0.9679; *p* < 0.0001) and pAb-cELISA (AUC = 0.9629; std. error = 0.008358; 95% CI = 0.9465 to 0.9793; *p* < 0.0001) (Figure 1C). FMDV/O mAb-bELISA demonstrated species-independent 98.93% DSe in contrast to 76.84% DSe (mAb-cELISA) and 80.92% DSe (pAb-cELISA) (Figure 1D; Table 2). Although a 50% cut-off was set as optimal for the species-independent assay, a slightly lower cut-off (30%) for porcine sera was used to obtain a 100% DSe, which was similar to mAb-cELISA (DSe = 100%) and pAb-cELISA (DSe = 100%) (Figure 1E; Table 2). At the 50% cut-off, the assay had a 100% DSe in bovine and ovine sera, which was higher than mAb-cELISA (DSe = 78.18%) and pAb-cELISA (DSe = 80.71%) (Figure 1F,G; Table 2).

The cross-reactivity analysis of FMDV/O mAb-bELISA in sera collected from animals experimentally infected with other FMDV serotypes and other vesicular disease viruses (swine vesicular disease [SVD], vesicular stomatitis virus [VSV], and Senecavirus A [SVA]) exhibited PI values below the set 30% and 50% cut-off for porcine (Figure 1H) and bovine (Figure 1I), respectively, except for one sample in bovine VSV/IND. This bovine VSV/IND serum sample was further examined by VNT and was found to have a negative (<16) serum antibody titer (Table S1). Thus, FMDV/O mAb-bELISA is assumed to be specific for the detection of antibodies against FMDV serotype O. 

### 3.2. DSp and DSe of FMDV/SAT2 mAb-bELISA

**Figure 2:**
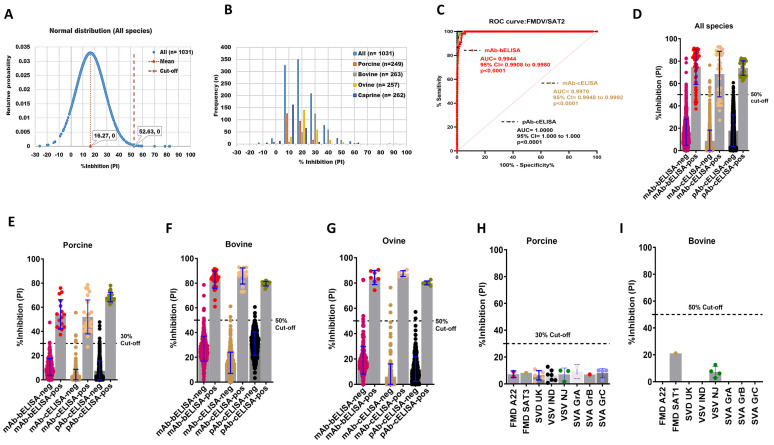
DSp and DSe of FMDV/SAT2-specific mAb-bELISA and comparator ELISAs. (**A**) Normal distribution of PI obtained from negative serum samples of Canadian healthy animal species (n = 1031). (**B**) Frequency distribution of PI in naïve negative animal sera (n = 1031 [all species], n = 249 [porcine], n = 263 [bovine], n = 257 [ovine], and n = 262 [caprine]). (**C**) ROC curve diagnostic sensitivity analysis of FMDV/SAT2 mAb-bELISA compared with mAb-cELISA and pAb-cELISA. (**D**–**G**) Bar graphs show the estimated PI cut-off line and overall DSe of FMDV/SAT2 mAb-bELISA and comparator ELISAs for all species (**D**), porcine (**E**), bovine (**F**), and ovine (**G**). (**H**,**I**) Cross-reactivity analysis of FMDV/SAT2 mAb-bELISA using sera from experimentally infected animals, such as porcine (**H**) and bovine (**I**), with other FMDV serotypes and other vesicular diseases. In (**A**), the red vertical dashed line and orange vertical dashed line represent estimated PI cut-off and mean values, respectively. Dashed lines in all bar graphs indicate the estimated PI cut-off.

Next, the mAb-bELISA was developed for the serodiagnosis of FMDV/SAT2 in different animal species. The goal was to develop this assay to have equal or improved diagnostic performance compared with conventional pAb-cELISA. This assay also generated a normal distribution of the frequencies of PI with an average 52.63% cut-off (Figure 2A,B), ranging from 32.04% to 56.90% (Figure S2A–D; Table 3). SAT2-specific mAb-bELISA scored a species-independent 98.55% DSp at a 50% cut-off (Table 3). A 100%, 98.10%, 98.05%, and 98.09% DSp were exhibited in porcine (n = 249), bovine (n = 263), ovine (n = 257), and caprine (n = 262) sera respectively (Table 3).

The DSe of FMDV/SAT2 mAb-bELISA was determined using sera from experimentally infected, contact-exposed, and vaccinated-challenged animals (porcine, bovine, and ovine) from different time points. The ROC curve analysis displayed an optimal DSe of mAb-bELISA (AUC = 0.9944; std. error = 0.001850; 95% CI = 0.9908 to 0.9980; *p* < 0.0001) in comparison with mAb-cELISA (AUC = 0.9970; std. error = 0.001117; 95% CI = 0.9948 to 0.9992; *p* < 0.0001) and pAb-cELISA (AUC = 1.000; std. error = 0.000; 95% CI = 1.000 to 1.000; *p* < 0.0001) (Figure 2C). More specifically, the assay showed a species-independent DSe of 87.50% compared with 69.56% (mAb-cELISA) and 100% (pAb-cELISA) (Figure 2D; Table 3). Interestingly, SAT2 mAb-bELISA demonstrated a 100% DSe in porcine sera at a 30% cut-off (Figure 2E), although it was somewhat lower at a 50% cut-off. A 100% DSe of mAb-bELISA was found in the sera of bovine and ovine with a 50% cut-off (Figure 2F,G, Table 3), which was similar to the mAb-cELISA and pAb-cELISA (Table 3).

Cross-reactivity analysis of SAT2-specific mAb-bELISA further exhibited a 100% DSp in the sera from porcine (30% cut-off) and bovine (50% cut-off) experimentally infected with other FMDV serotypes (Figure 2H,I). These data demonstrate that the FMDV/SAT2 mAb-bELISA is specific against anti-FMDV/SAT2 antibodies.

### 3.3. DSp and DSe of FMDV/A mAb-bELISA

**Figure 3:**
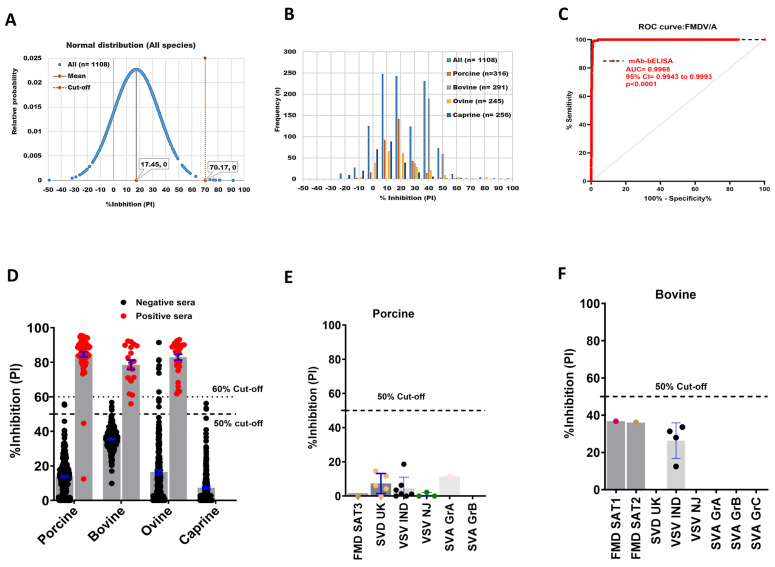
DSp and DSe of FMDV/A-specific mAb-bELISA. (**A**) Normal distribution of PI recorded from negative serum samples of Canadian healthy animal species (n = 1108). (**B**) Frequency distribution of PI in negative animal sera (n = 1108 [all species], n = 316 [porcine], n = 291 [bovine], n = 245 [ovine], and n = 256 [caprine]). (**C**) ROC curve diagnostic sensitivity analysis of FMDV/A mAb-bELISA (**D**) Bar graph shows the estimated PI cut-off line (either 50% or 60%) and overall DSe of FMDV/A mAb-bELISA for porcine, bovine, and ovine. No positive sera were tested for caprine. (**E**,**F**) Cross-reactivity analysis of FMDV/A mAb-bELISA with porcine (**E**) and bovine (**F**) sera experimentally infected with other FMDV serotypes and other vesicular diseases. In (**A**), the red vertical dashed line and grey vertical solid line represent estimated PI cut-off and mean values, respectively. Dashed lines in all bar graphs indicate the estimated PI cut-off.

An FMDV/A mAb-bELISA was developed using mouse anti-FMDV/A mAb. A total of 1108 serum samples collected from negative animals, including porcine (n = 316), bovine (n = 291), ovine (n = 245), and caprine (n = 256) were examined. Figure 3A,B shows the normal distribution and frequencies of PI in negative animal sera, with an overall 70.17% cut-off, ranging between 45.28% and 74.74% (Figure S3A–D). Because porcine, bovine, and caprine sera demonstrated a relatively close cut-off range of approximately 50%, we applied an arbitrary 50% cut-off for this assay, which provided a 98.01% DSp (Table 4). Importantly, the assay exhibited a higher DSp for porcine (99.37%), bovine (98.97%), and caprine (98.82%) compared with that of ovine sera (94.33%) (Table 4). 

The ROC curve analysis revealed an optimal DSe of FMDV/A mAb-bELISA in serially bled sera of known FMDV/A infected and vaccinated-challenged animals (AUC = 0.9968; std. error = 0.001267; 95% CI = 0.9943 to 0.9993; *p* < 0.0001) (Figure 3C). With a 50% cut-off, the assay indicated a species-independent 98.25% DSe (Table 4). The mAb-bELISA also exhibited a 100% DSe in the sera of bovine and ovine, and 96.88% for porcine (Figure 3D; Table 4), in which a 98.44% DSe could be achieved with a cut-off set at 40% (Table 4). Two porcine-positive sera showed PI values less than the 50% cut-off, which was further confirmed by VNT. The results from VNT revealed that these two porcine sera were negative (<16 sera antibody titers) (Table S1), supporting the data generated by bELISA. Positive sera used for this study originated from animals infected with either the FMDV A22 Iraq 24/64 or the A24 Cruzeiro/Br/55 strains. Interestingly, FMDV/A antigens and mAb used in the design of the FMDV/A bELISA were able to detect positive antibodies against either strain.

Serotype A-specific mAb-bELISA further indicated a 100% analytical specificity in cross-reaction analysis with serum samples from animals known to be experimentally infected with other FMDV serotypes (Figure 3E,F).

### 3.4. DSp and DSe of FMDV/Asia1 mAb-bELISA 

**Figure 4:**
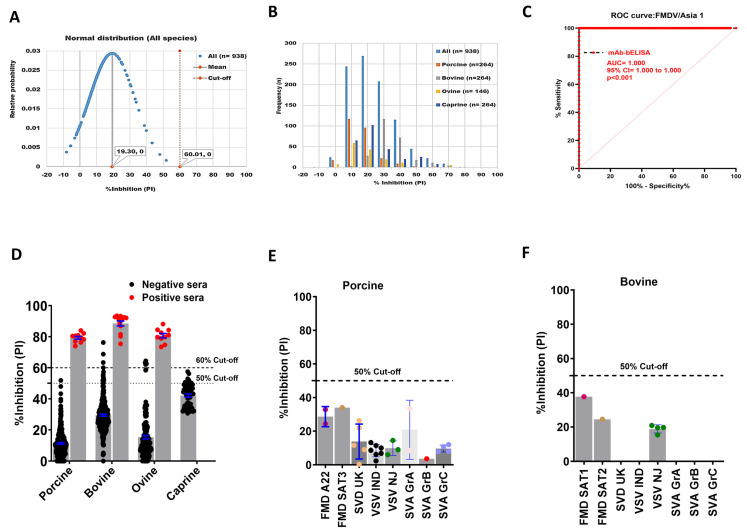
DSp and DSe of FMDV/Asia1-specific mAb-bELISA. (**A**) Normal distribution of PI obtained from negative serum samples of Canadian healthy animal species (n = 938). (**B**) Frequency distribution of PI in negative animal sera (n = 938 [all species], n = 264 [porcine], n = 254 [bovine], n = 146 [ovine], and n = 264 [caprine]). (**C**) ROC curve diagnostic sensitivity analysis of FMDV/Asia1 mAb-bELISA. (**D**) Bar graph shows the estimated PI cut-off line (either 50% or 60%) and overall DSe of FMDV/Asia1 mAb-bELISA for porcine, bovine, and ovine. No positive sera were tested for caprine. (**E**,**F**) Cross-reactivity analysis of FMDV/A mAb-bELISA with porcine (**E**) and bovine (**F**) sera experimentally infected with other FMDV serotypes and other vesicular diseases. In (**A**), the red vertical dashed line and grey vertical solid line represent estimated PI cut-off and mean values, respectively. Dashed lines in all bar graphs indicate the estimated PI cut-off.

A serotype-specific mAb-bELISA was developed for the detection of antibodies against FMDV/Asia1. Similarly, the assay demonstrated a normal distribution and frequencies of PI with an average 60.01% cut-off (Figure 4A,B); the upper and lower limits ranged from 37.79% (porcine) to 61.79% (bovine), respectively (Figure S4A–D). An arbitrary 50% cut-off was further applied to this assay, which revealed a 96.59% DSp in known negative animal sera (n = 938), including porcine (n = 264), bovine (n = 264), ovine (n = 146), and caprine (n = 264) (Table 5).

The DSe of the assay was also analyzed by ROC curve analysis, revealing an optimal species-independent diagnostic sensitivity (AUC = 1.000; std. error = 0.0000; 95% CI = 1.000 to 1.000; *p* < 0.0001) (Figure 4C). Using a 50% cut-off, FMDV/Asia1 mAb-bELISA exhibited a 100% DSe for all animal sera examined (Figure 4D). Cross-reactivity analysis again showed that the mAb-based Asia1-specific bELISA was specific against anti-FMDV/Asia1 antibodies (Figure 4E,F).

### 3.5. Detection Efficiency of FMDV Serotype-Specific mAb-bELISAs

**Figure 5:**
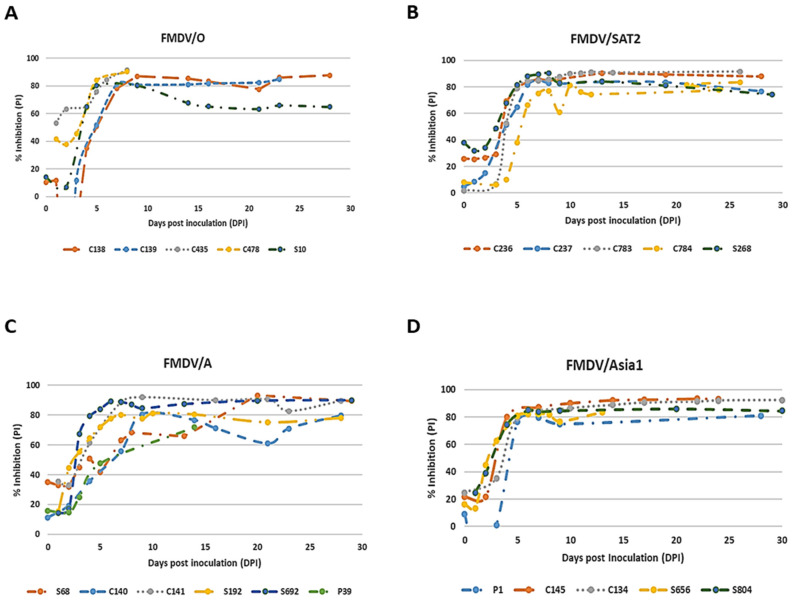
Detection efficiency of FMDV serotype-specific mAb-bELISAs. Pigs, cows, and sheep were experimentally infected with FMDV serotypes O/Manisa, SAT2/SAU, A22/Iraq, and Asia1/Shamir. Serially bled sera were collected at 0–30 dpi and examined by using FMDV/O mAb-bELISA (**A**), FMDV/SAT2 mAb-bELISA (**B**), FMDV/A mAb-bELISA (**C**), and FMDV/Asia1 mAb-bELISA (**D**). p—pig; c—cow; s—sheep.

To determine how quickly the serotype-specific mAb-bELISAs detect anti-FMDV antibodies, the seroconversion kinetics were determined using serially bled sera collected at different dpi from experimentally infected, contact exposure, and vaccination-challenged animals. The mAb-bELISAs detected anti-FMDV antibodies as early as 5 dpi with a 50–60% cut-off (Figure 5A–D). However, early time point data (0–9 dpi) demonstrated relatively low PIs (below serotype-specific cut-off) for most of the contact-exposed animal sera, indicating a negative antibody response. The negative sera were further confirmed by VNT. Thus, sera from approximately 10 dpi were considered for calculating the relative DSe of the assays.

### 3.6. Repeatability/Reproducibility of the FMDV Serotype-Specific mAb-bELISAs

The repeatability/reproducibility was evaluated for all four FMDV serotype-specific mAb-bELISAs using a serotype-specific weak positive control sera panel. The assays exhibited a % coefficient of variation (CV) of 4.08%, 2.37%, 7.21%, and 1.67% for serotypes O, SAT2, A, and Asia1, respectively, regarding inter-plates (Table 6). In the case of intra-plates, the assays recorded 1.46%, 1.64%, 2.58%, and 0.87% CV for serotypes O, SAT2, A, and Asia1, respectively (Table 6). Therefore, all four assays exhibited %CV within the set range, which was <15% for inter-plates and <10% for intra-plates. These data suggest a high degree of repeatability/reproducibility of all in-house developed mAb-bELISAs for FMDV serotypes.

## 4. Discussion

FMDV-infected ruminants, particularly buffalos, cattle, and sheep, develop a carrier state, and carrier African buffalos can act as a new source of infection and disease outbreaks [32]. It is therefore critical to identify infected and/or carrier state animals for an effective control strategy to limit the spread of the virus. Vaccination of animals with inactivated FMDV vaccines is currently considered the primary means of controlling FMD worldwide [14,15,16,33]. However, the protection efficiency of the FMDV vaccines is relatively short (~6 months) and requires regular monitoring of serotype-specific antibodies to re-vaccinate animals following an initial dose [34,35]. The VNT is used as a “gold standard” serological assay for measuring neutralizing antibody titers against FMDV serotypes [31]. Unfortunately, the VNT is labor-intensive, difficult to use on a large scale, highly dependent on containment facilities, time-consuming, and has a long turnaround time [7]. In contrast, the application of ELISA for the detection of serotype-specific FMDV antibodies is faster because it does not require cell culture systems and high containment facilities [36]. Thus, a convenient, safe, reproducible, and serotype-specific FMD screening ELISA for large-scale sero-surveillance of broad-range antibodies, is warranted to minimize diagnostic inaccuracies and turnaround time where VNT could still serve as a “gold standard” confirmatory test. It has been reported that ELISAs performed with 97% to 98% DSp are capable of detecting carrier states in vaccinated animals and infected animals with DSe ranges between 68% and 94% [37,38].

Since the early phase of research and diagnosis of FMDV, pAbs or antiserum have been used as primary tools in developing serotype-specific diagnostic ELISAs [24]. Due to drawbacks of the large-scale use of pAbs [1,39,40,41], the use of mAbs to replace pAbs was evaluated to develop FMDV serotype-specific bELISAs in this study. Our primary goal was to establish mAb-based bELISAs that would provide better or equivalent assay performance compared with pAb-based competitive ELISA (pAb-cELISA) and to replace the use of pAbs in serotype-specific FMDV ELISAs in the future. In this study, four mAb-based FMDV bELISAs were developed for the serodiagnosis of O, A, Asia1, and SAT2 serotypes and monitoring of antibody titers against these serotypes following vaccination. 

FMDV/O is highly prevalent and causes nearly 70% of global outbreaks [8,42]. Therefore, an FMDV/O mAb-bELISA using an inactivated FMDV/O Manisa antigen was developed. The FMDV/O mAb-bELISA showed a species-independent 99.74% DSp and 98.93% DSe in all sera examined (at 50% cut-off). The ROC curve analysis indicated an optimal DSe of the assay in detecting antibodies against FMDV/O among infected and vaccinated animals compared with mAb-cELISA and pAb-cELISA. This serotype O mAb-bELISA performed better than the previously reported assays [21,29]. In addition, a higher diagnostic specificity and sensitivity of mAb-bELISA in individual animal species (100% DSp for porcine, ovine, and caprine sera and 100% DSe for bovine and ovine sera) was observed in contrast to the assay reported by Paiba et al. [22]. A study on mAb-based ELISA also recently demonstrated serotype-specific 79% sensitivity in comparison with pAb-based ELISA with 72% sensitivity [41]. Using VLPs (virus-like particles) and rabbit hyperimmune sera, another study showed 96% assay sensitivity [43]. Importantly, a 50% cut-off was considered ideal for separating true positive and true negative populations in our assay; however, a slightly lower cut-off further improved the DSe for porcine sera (100% DSe at a 30% cut-off; Table 1). Although a 50% cut-off seemed to be ideal for bovine sera (100% DSe); two samples showed a PI value above the set cut-off, leaving a 99.24% DSp without interfering with DSe. Finally, our assay demonstrated a better DSe for ovine sera compared with pAb-cELISA and the cross-reactivity analysis showed that the assay was specific to FMDV serotype O. 

An FMDV/SAT2-specific mAb-bELISA was developed. FMDV/SAT viruses are mainly circulating and restricted in the wildlife of sub-Saharan Africa, particularly in African buffalo, where they establish a persistent infection [44]. Few studies have been performed in developing and validating SAT2-specific ELISA [45,46,47,48]. A solid-phase competitive ELISA (SPCE) for FMDV/SAT2 using sera from cattle herds showed only 81% DSe (at 50% cut-off), and 100% DSp while the cut-off was set at 60% [48]. However, a rapid and more sensitive ELISA to detect anti-SAT2 antibodies in large-scale samples is still desirable. The SAT2 mAb-bELISA developed in this study exhibited a species-independent 98.55% DSp and 87.5% DSe (at a 50% cut-off). It has also been shown that a high cut-off usually increases the DSp while decreasing the DSe and vice versa [21]. Thus, an arbitrary 40% cut-off was applied to achieve a minimum DSe of the assay (at least 95%). With an adjusted 40% cut-off, we found a species-independent 98.21% DSe and 96.22% DSp (Table 3). In addition, the source of sera demonstrated some impact on DSp and DSe in our assay. We observed a 100% DSe in bovine and ovine sera, while only 56.25% was recorded in porcine sera (at 50% cut-off). This is not an indication of the assay’s inability to provide optimal DSe in porcine sera, rather it highlights the relative sensitivity of the assay in detecting antibodies against SAT2 with a low cut-off margin (~30%), since most of the negative porcine sera stayed below the 30% cut-off. As a result, a 30% cut-off is important to achieve 100% DSe for porcine sera (Table 3). It is also important to note that lowering the cut-off to 30% was only needed for the porcine sera and was not likely essential for bovine and ovine sera. Furthermore, mAb-bELISA scored a similar DSe to pAb-cELISA, suggesting that it can be used as a replacement for FMDV/SAT2-specific pAb-cELISA.

The FMDV/A serotype is present in most regions with other FMDVs and is establishing many new subtypes [49]. The mAb-bELISA for FMDV/A was developed with an optimal diagnostic sensitivity found in ROC curve analysis (AUC = 0.9968; *p* < 0.0001). Using 1108 known negative sera of different origins, this assay displayed an average 70.17% cut-off, ranging between 45.28% and 74.74%. Exceptionally high PI values were recorded for 14 ovine-negative sera (at 50% cut-off) causing an overall high baseline for species-independent assay. Therefore, we also applied an arbitrary cut-off of 50% (22 samples were above the cut-off), resulting in a species-independent 98.01% DSp and 98.24% DSe to detect antibodies against FMDV/A. Interestingly, this assay showed better diagnostic performance than the assay developed by Hosamani et al. [29], who reported assay specificity and sensitivity of 98% and 95%, respectively. The assay developed by Grazioli et al. [41] also recorded only 79% specificity. Two porcine sera were recorded below the cut-off line in our assay, which were confirmed as true negatives by VNT (Table S1). Removing these two porcine sera from the analysis would provide 100% DSe for a species-independent assay, which corroborates the assay developed by Cao et al. [50]. Additionally, the FMDV/A mAb-bELISA is found to be specific for serotype A according to the cross-reactivity analysis. 

Finally, an FMDV/Asia1-specific mAb-bELISA was developed. Serotype Asia1 mainly circulates in Asia, with sporadic outbreaks in Europe and the Middle East [41]. The Asia1 mAb-bELISA demonstrated a species-independent DSp of 96.59% (cut-off at 50%) or 98.93% (cut-off at 60%) in all examined negative animal sera with a DSe of 100%. No changes in DSe were observed when the cut-off was switched from 50% to 60%, which was further supported by ROC curve analysis, showing an optimal diagnostic sensitivity (AUC: 1.000; *p* < 0.0001). Although the assay identified a cut-off range between 37.79% (porcine) and 61.79% (bovine), a 60% cut-off would be an ideal choice for species-independent use of this assay against FMDV/Asia1 antibodies. Our FMDV/Asia1 mAb-bELISA had better diagnostic performance than previously defined assays [5,41]. A recent multiplex mAb-ELISA for serotypes O, A, C, and Asia1 reported only 79% sensitivity [41], which was much lower than our assay. Cross-reactivity analysis further exhibited a 100% specificity of our assay against FMDV/Asia1 antibodies.

The mAb-bELISAs developed in this study successfully detected antibody response in serially bled sera as early as 5 dpi, with a relatively high ratio of negative results until 9 dpi. Sera showing negative results were mostly collected from contact-exposed animals in transmission studies between 5 and 9 dpi, where lack of antibody detection by serotype-specific mAb-bELISAs suggested that these animals were most likely not infected by the test viruses or had undetectable levels of antibody response. Therefore, up to 9 dpi can be considered a critical zone, and 10 dpi and above could be ideal for a strong antibody response to be detected by our assays. Several studies have also shown that approximately 10 dpi sera were perfect for actual positivity detection by ELISA [26,37,51,52]. In addition, all our assays showed a strong repeatability/reproducibility by producing %CVs within the set assay range for inter-plate and intra-plate assay variabilities (Table 6).

## 5. Conclusions

In summary, all four novel mAb-based FMDV serotype-specific ELISAs described herein particularly demonstrated high diagnostic performance (Table 7). These ELISAs were validated using a reasonably large number of serum samples from different origins, which proved and confirmed that serotype-specific mAb-bELISAs with a relatively shorter turnaround time can be used as a reliable diagnostic tool to replace existing pAb-based FMDV ELISAs. These mAb-ELISAs would be useful for large-scale sero-surveillance of FMDV serotypes and monitoring of serotype-specific vaccine effectiveness to control FMD globally. In future studies, mAb-bELISAs, where inactivated FMDV antigens are used, will be replaced by VLPs, which would particularly enhance the safe use of serotype-specific mAb-bELISA without high containment facilities.

## Figures and Tables

**Table 1:** Serotype-specific reagents.

FMDV Serotype	Capture Antibody/Dilution	Antigen/Dilution	Q1/Dilution	Q2/Dilution	Q3/Dilution	mAb/Dilution
A	α-FMDV A22/Iraq 24/64 (1:10,000)	A22 Iraq 24/64 (1:75)	C141 A24/CRUZ (undiluted)	C141 A24/CRUZ (1:10)	NBS	F66-A22-14 (1:2000)
Asia1	α-FMDV Asia1/Shamir (1:14,000)	Asia1/Shamir (1:300)	C145 Asia1/Shamir (undiluted)	C145 Asia1/Shamir (1:50)	NBS	F34-Asia1 (1:2000)
SAT2	α-FMDV SAT2/Zim10/91 (1:1000)	SAT2/SAU1/2000 (1:100)	C783 SAT2/ZIM 5/81 (undiluted)	C783 SAT2/ZIM 5/81 (1:10)	NBS	F76-SAT2-11-2-1 (1:1500)

NBS—normal bovine serum.

**Table 2:** Diagnostic specificity and sensitivity of mAb-bELISA to detect antibodies against FMDV/O and other comparator ELISAs.

Animal Species	PI Cut-Off	mAb-bELISA		mAb-cELISA		pAb-cELISA	
		DSp	DSe	DSp	DSe	DSp	DSe
		Naïve Animals	Infected Animals	Naïve Animals	Infected Animals	Naïve Animals	Infected Animals
All	30%	89.21% (678/760)	100% (99/99)	95.00% (1263/1329)	88.71% (253/285)	98.00% (1273/1298)	91.87% (260/283)
	40%	97.89% (744/760)	98.93% (98/99)	98.65% (1311/1329)	83.86% (239/285)	99.69% (1294/1298)	88.34% (250/283)
	50%	99.74% (758/760)	98.93% (98/99)	100% (1329/1329)	76.84% (219/285)	99.85% (1296/1298)	80.92% (229/283)
	60%	100% (760/760)	90.00% (89/99)	100% (1329/1329)	69.47% (198/285)	99.92% (1297/1298)	73.14% (207/283)
Porcine	30%	100% (262/262)	100% (13/13)	100% (535/535)	100% (29/29)	99.63% (533/535)	100% (29/29)
	40%	100% (262/262)	92.31% (12/13)	100% (535/535)	86.21% (25/29)	100% (535/535)	100% (29/29)
	50%	100% (262/262)	92.31% (12/13)	100% (535/535)	79.31% (23/29)	100% (535/535)	89.66% (26/29)
	60%	100% (262/262)	61.54% (8/13)	100% (535/535)	41.38% (12/29)	100% (535/535)	75.86% (22/29)
Bovine	30%	70.08% (185/264)	100% (29/29)	88.18% (470/533)	-	95.87% (511/533)	100% (20/20)
	40%	94.32% (249/264)	100% (29/29)	96.81% (516/533)	-	95.87% (511/533)	100% (20/20)
	50%	99.24% (262/264)	100% (29/29)	100% (533/533)	-	99.62% (531/533)	90% (18/20)
	60%	100% (264/264)	96.55% (28/29)	100% (533/533)	-	99.81% (532/533)	70% (14/20)
Ovine	30%	98.74% (157/159)	100% (57/57)	98.85% (258/261)	87.50% (224/256)	99.13% (228/230)	90.94% (231/254)
	40%	99.37% (158/159)	100% (57/57)	99.62% (260/261)	83.98% (215/256)	99.13% (228/230)	87.01% (221/254)
	50%	100% (159/159)	100% (57/57)	100% (261/261)	78.13% (200/256)	100% (230/230)	80.71% (205/254)
	60%	100% (159/159)	94.74% (54/57)	100% (261/261)	75.39% (193/256)	100% (230/230)	73.62% (187/254)
Caprine	30%	100% (75/75)	-	-	-	-	-
	40%	100% (75/75)	-	-	-	-	-
	50%	100% (75/75)	-	-	-	-	-
	60%	100% (75/75)	-	-	-	-	-

**Table 3:** Diagnostic specificity and sensitivity of mAb-bELISA to detect antibody against FMDV/SAT2 and other comparator ELISAs.

Animal Species	PI Cut-Off	mAb-bELISA		mAb-cELISA		pAb-cELISA	
		DSp	DSe	DSp	DSe	DSp	DSe
		Naïve Animals	Infected Animals	Naïve Animals	Infected Animals	Naïve Animals	Infected Animals
All	30%	88.65% (914/1031)	100% (56/56)	96.78% (1293/1336)	97.83% (45/46)	76.67% (1022/1333)	100% (46/46)
	40%	96.22% (992/1031)	98.21% (55/56)	99.03% (1323/1336)	95.65% (44/46)	91.97% (1226/1333)	100% (46/46)
	50%	98.55% (1016/1031)	87.50% (49/56)	99.55% (1330/1336)	69.56% (32/46)	99.40% (1325/1333)	100% (46/46)
	60%	99.61% (1027/1031)	80.36% (45/56)	99.78% (1333/1336)	60.87% (28/46)	99.92% (1332/1333)	100% (46/46)
Porcine	30%	98.78% (246/249)	100% (16/16)	98.70% (533/540)	95.83% (23/24)	98.88% (532/538)	100% (24/24)
	40%	99.60% (248/249)	93.75% (15/16)	99.81% (539/540)	91.67% (22/24)	99.44% (535/538)	100% (24/24)
	50%	100% (249/249)	56.25% (9/16)	99.81% (539/540)	41.67% (10/24)	100% (538/538)	100% (24/24)
	60%	100% (249/249)	31.25% (5/16)	99.81% (539/540)	25.00% (6/24)	100% (538/538)	100% (24/24)
Bovine	30%	69.58% (183/263)	100% (33/33)	94.94% (507/534)	100% (16/16)	45.13% (241/534)	100% (16/16)
	40%	92.02% (242/263)	100% (33/33)	98.69% (527/534)	100% (16/16)	81.27% (434/534)	100% (16/16)
	50%	98.10% (258/263)	100% (33/33)	99.63% (532/534)	100% (16/16)	98.88% (528/534)	100% (16/16)
	60%	99.24% (261/263)	100% (33/33)	100% (534/534)	100% (16/16)	100% (534/534)	100% (16/16)
Ovine	30%	89.49% (230/257)	100% (7/7)	96.56% (253/262)	100% (14/14)	95.79% (250/261)	100% (14/14)
	40%	95.72% (246/257)	100% (7/7)	98.09% (257/262)	100% (14/14)	98.47% (257/261)	100% (14/14)
	50%	98.05% (252/257)	100% (7/7)	98.85% (259/262)	100% (14/14)	99.23% (259/261)	100% (14/14)
	60%	99.22% (255/257)	100% (7/7)	99.24% (260/262)	100% (14/14)	100% (261/261)	100% (14/14)
Caprine	30%	97.33% (255/262)	-	-	-	-	-
	40%	97.71% (256/262)	-	-	-	-	-
	50%	98.09% (257/262)	-	-	-	-	-
	60%	100% (262/262)	-	-	-	-	-

Since the data generated by FMDV serotypes O- and SAT2-specific mAb-bELISAs were closely comparable to that of mAb-cELISA and pAb-cELISA, serotypes A and Asia1 were not compared with cELISAs. Thus, the data on serotypes A and Asia1 presented in the following sections were solely generated by mAb-bELISAs.

**Table 4:** Diagnostic specificity and sensitivity of mAb-bELISA to detect antibodies against FMDV/A.

Animal Species	PI Cut-Off	mAb-bELISA	
		DSp	DSe
		Naïve Animals	Infected Animals
All	30%	67.24% (745/1108)	99.12% (113/114)
	40%	91.43% (1013/1108)	99.12% (113/114)
	50%	98.01% (1086/1108)	98.25% (112/114)
	60%	99.19% (1099/1108)	97.37% (111/114)
	70%	99.28% (1100/1108)	90.35% (103/114)
Porcine	30%	93.99% (297/316)	98.44% (63/64)
	40%	98.42% (311/316)	98.44% (63/64)
	50%	99.37% (314/316)	96.88% (62/64)
	60%	100% (316/316)	96.88% (62/64)
Bovine	30%	13.40% (39/291)	100% (18/18)
	40%	78.69% (229/291)	100% (18/18)
	50%	98.97% (288/291)	100% (18/18)
	60%	100% (291/291)	94.44% (17/18)
Ovine	30%	82.04% (201/245)	100% (32/32)
	40%	90.61% (222/245)	100% (32/32)
	50%	94.29% (231/245)	100% (32/32)
	60%	95.92% (235/245)	100% (32/32)
	70%	97.14% (238/245)	84.38% (27/32)
Caprine	30%	95.70% (245/256)	-
	40%	98.05% (251/256)	-
	50%	98.83% (253/256)	-
	60%	100% (256/256)	-

**Table 5:** Diagnostic specificity and sensitivity of mAb-bELISA to detect antibody against FMDV/Asia1.

Animal Species	PI Cut-Off	mAb-bELISA	
		DSp	DSe
		Naïve Animals	Infected Animals
All	30%	79.43% (746/938)	100% (33/33)
	40%	91.79% (861/938)	100% (33/33)
	50%	96.59% (906/938)	100% (33/33)
	60%	98.93% (928/938)	100% (33/33)
	70%	99.89% (937/938)	100% (33/33)
Porcine	30%	95.45% (252/264)	100% (10/10)
	40%	98.86% (261/264)	100% (10/10)
	50%	99.62% (263/264)	100% (10/10)
	60%	100% (264/264)	100% (10/10)
Bovine	30%	58.71% (155/264)	100% (13/13)
	40%	87.12% (230/264)	100% (13/13)
	50%	93.94% (248/264)	100% (13/13)
	60%	98.11% (259/264)	100% (13/13)
Ovine	30%	87.67% (128/146)	100% (10/10)
	40%	95.21% (139/146)	100% (10/10)
	50%	95.21% (139/146)	100% (10/10)
	60%	96.58% (141/146)	100% (10/10)
	70%	100% (146/146)	100% (10/10)
Caprine	30%	79.92% (211/264)	-
	40%	87.50% (231/264)	-
	50%	96.97% (256/264)	-
	60%	100% (264/264)	-

**Table 6:** Repeatability/reproducibility of the FMDV serotype-specific mAb-bELISAs.

Variability between Plates					Variability within Plates				
Serotypes	n	Mean PI	Stdev	CV%	Serotypes	n	Mean PI	Stdev	CV%
O	23	70.32	2.87	4.08	O	24	70.72	1.03	1.46
SAT2	30	78.91	1.87	2.37	SAT2	26	82.95	1.36	1.64
A	35	69.41	5.00	7.21	A	26	69.37	1.79	2.58
Asia1	26	86.17	1.44	1.67	Asia1	26	82.39	0.72	0.87

n—number of plates, PI—percent inhibition, stdev—standard deviation, CV—coefficient of variation.

**Table 7:** Final PI cut-off, DSp, and DSe of species-independent FMDV serotype-specific mAb-bELISA.

FMDV Serotypes	PI Cut-Off	mAb-bELISA	
		DSp	DSe
O	50%	99.74%	98.93%
SAT2	40%	96.22%	98.21%
A	50%	98.01%	98.25%
Asia1	50%	96.59%	100%

## Data Availability

All data supporting the findings of this study are available from the corresponding author upon a reasonable request.

## Supplementary Materials

The following supporting information can be downloaded at: https://www.mdpi.com/article/10.3390/v16091438/s1, Table S1: Serum antibody titer generated by VNT; Figure S1. Distribution of PI values recorded by FMDV/O-specific mAb-bELISA; Figure S2. Distribution of PI values recorded by FMDV/SAT2-specific mAb-bELISA; Figure S3. Distribution of PI values recorded by FMDV/A-specific mAb-bELISA; Figure S4. Distribution of PI values recorded by FMDV/Asia1-specific mAb-bELISA.
