# Supplementary material for: Development and Validation of Serotype-Specific Blocking ELISA for the Detection of Anti-FMDV O/A/Asia1/SAT2 Antibodies
Source: Viruses. 2024 Sep 10;16(9):1438. doi: 10.3390/v16091438 (PMC11437413; doi:10.3390/v16091438)
Supplement: Supplementary file 1 [file viruses-16-01438-s001.zip › viruses-3176254-supplementary.pdf]

Supplemental table and figures

Article

Development and Validation of Serotype-Specific Blocking ELISA for the Detection of Anti-FMDV O/A/Asia1/SAT2 Antibodies

Mohammad A. Kashem, Patrycja Sroga, Vivien Salazar, Hamza Amjad, Kate Hole, Janice Koziuk, Ming Yang, Charles Nfon and Shawn Babiuk *

National Centre for Foreign Animal Disease, Canadian Food Inspection Agency, Winnipeg, MB R3E 3M4, Canada; mohammad.kashem@inspection.gc.ca (M.A.K.); patrycja.sroga@inspection.gc.ca (P.S.); vivien.salazar@inspection.gc.ca (V.S.); hamza.amjad@inspection.gc.ca (H.A.); kate.hole@inspection.gc.ca (K.H.); janice.koziuk@inspection.gc.ca (J.K.); ming.yang@inspection.gc.ca (M.Y.); charles.nfon@inspection.gc.ca (C.N.)

* Correspondence: shawn.babiuk@inspection.gc.ca; Tel.: +1-204-784-5956

Table S1: Serum antibody titer generated by VNT

Animals and viral strains	Antibody titer	Assay used
Bovine VSV/IND	<16	FMDV/O mAb-bELISA
Porcine FMDV/A24 Cruz, DPI 14	<16	FMDV/A mAb- bELISA
Porcine FMDV/A22, DPI 17	<16	FMDV/A mAb- bELISA

VSV/IND, vesicular stomatitis virus/Indiana; Cruz, cruzeiro; DPI, days post inoculation

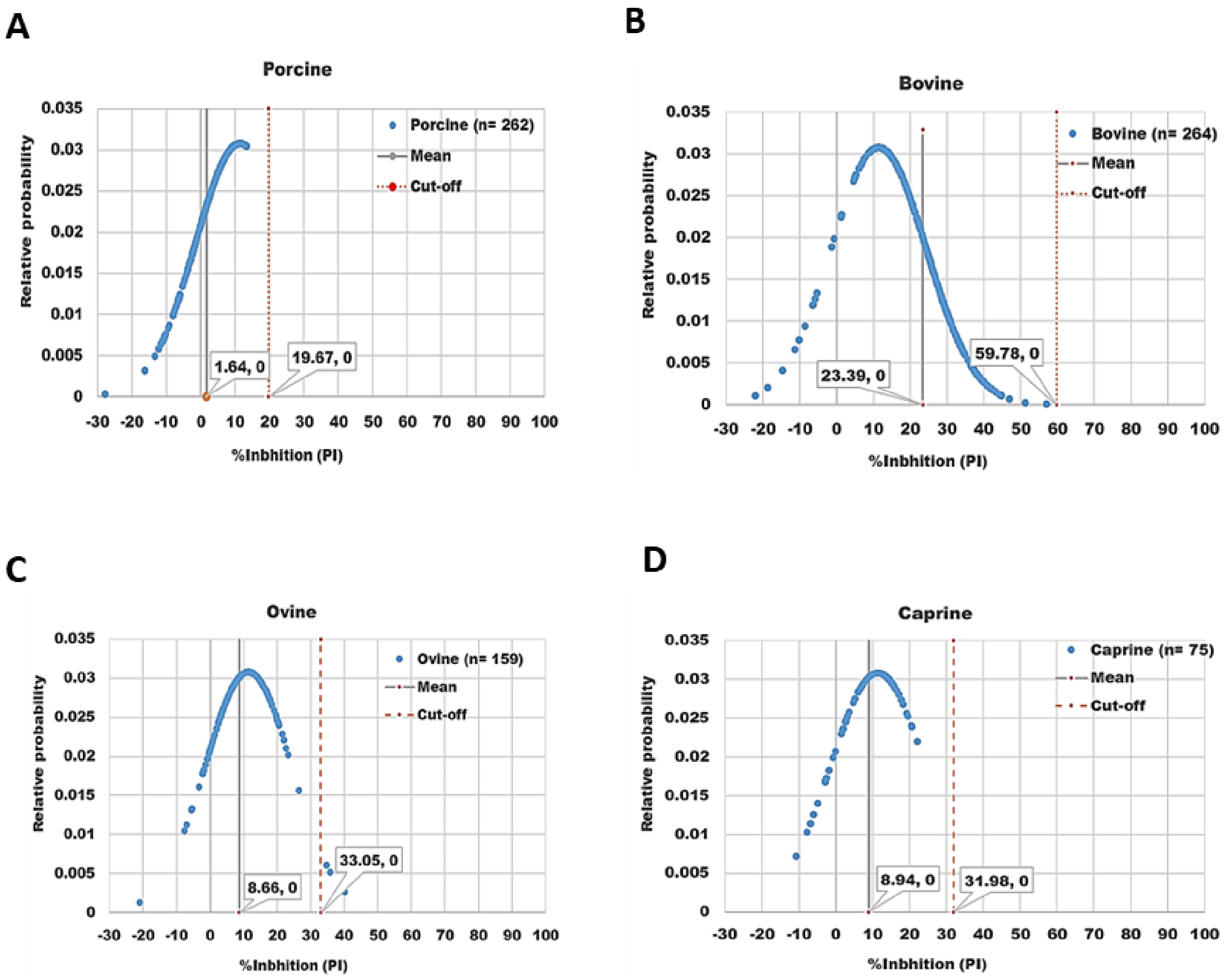

Figure S1. Distribution of PI values recorded by FMDV/O-specific mAb-bELISA. Normal distribution of PI obtained from naïve negative sera of Canadian healthy porcine (A), bovine (B), ovine (C), and caprine (D). The red vertical dashed line and grey vertical solid line represent estimated PI cut-off and mean values, respectively.

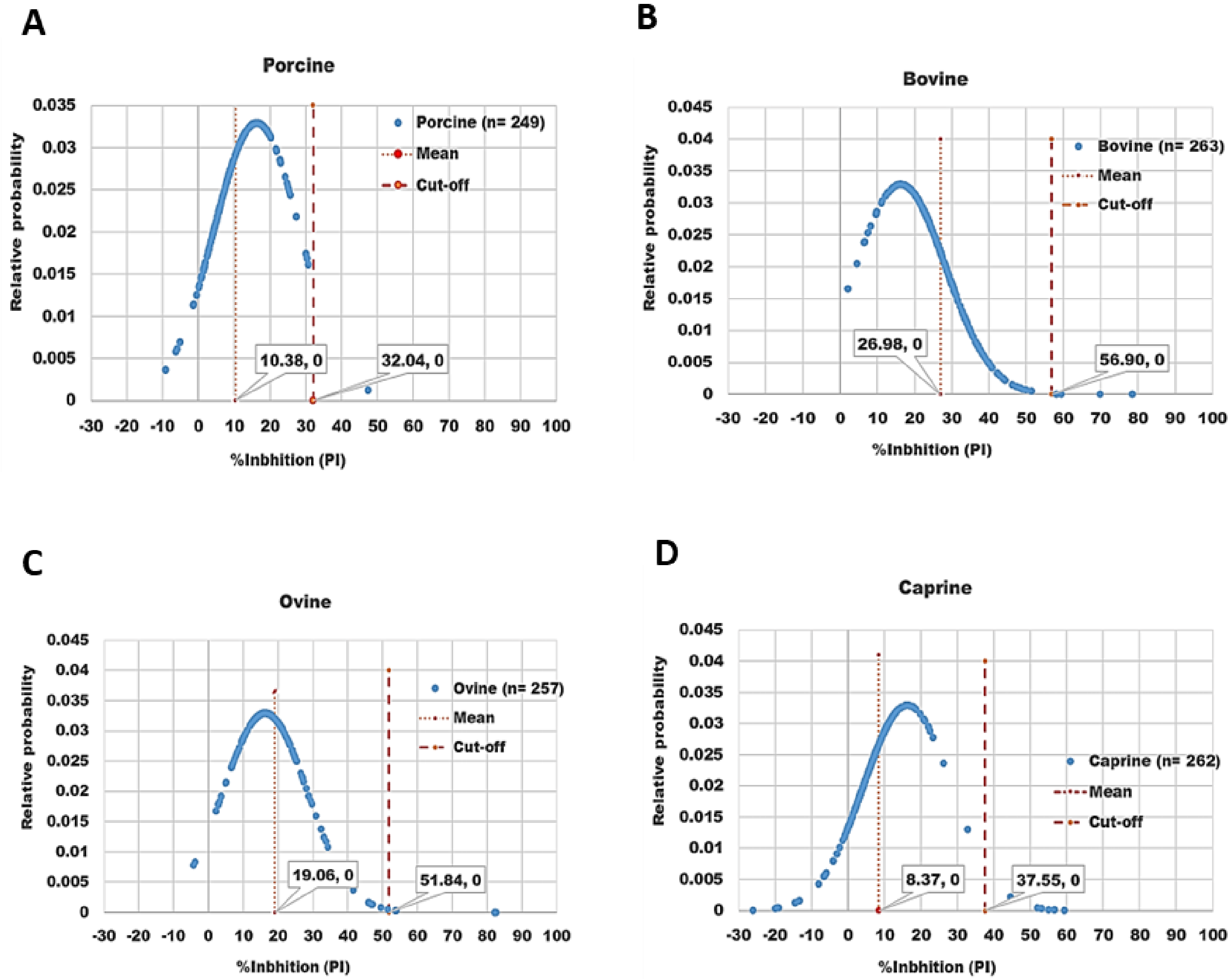

Figure S2. Distribution of PI values recorded by FMDV/SAT2-specific mAb-bELISA. Normal distribution of PI obtained from naïve negative sera of Canadian healthy porcine (A), bovine (B), ovine (C), and caprine (D). The red vertical dashed line and grey vertical solid line represent estimated PI cut-off and mean values, respectively.

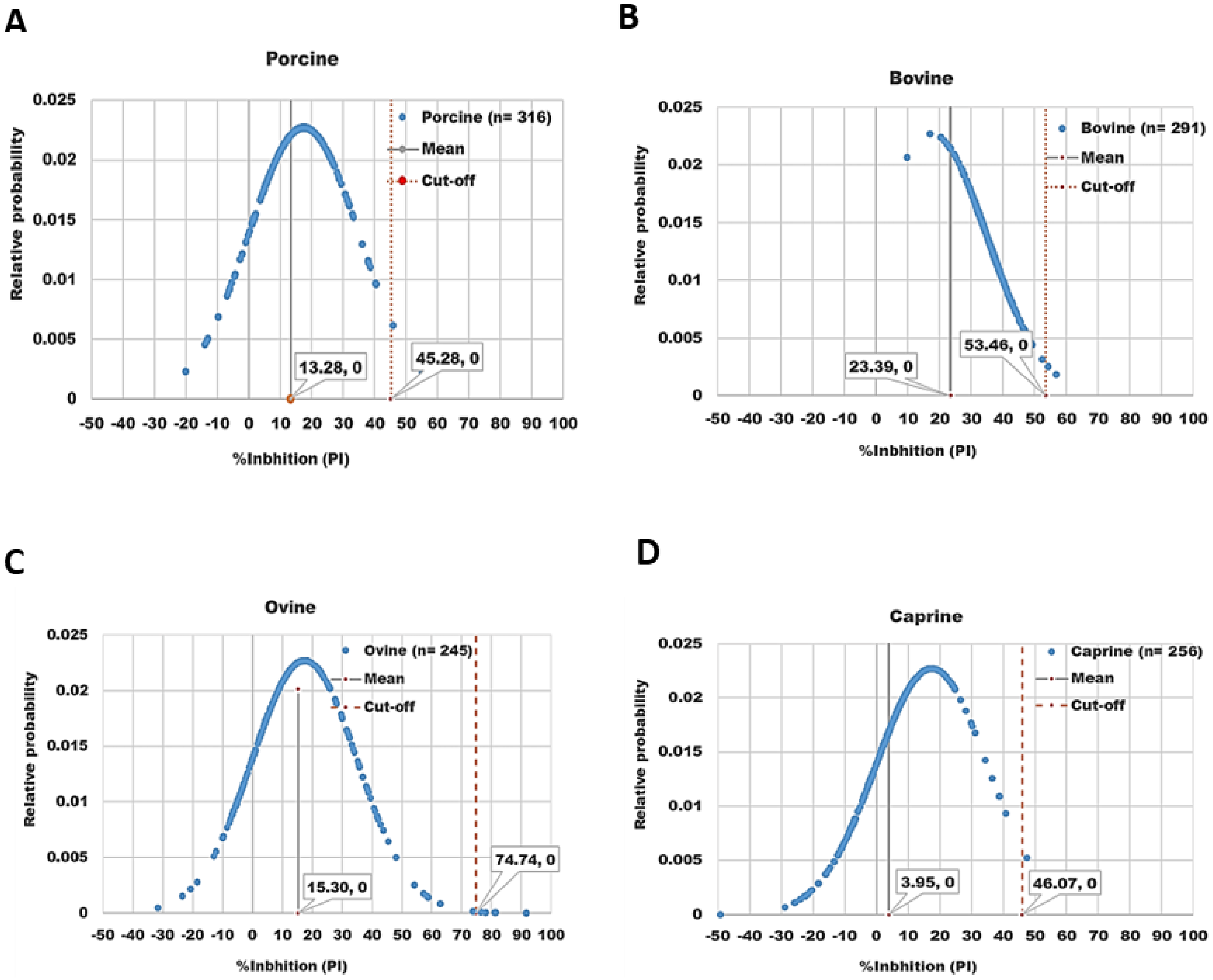

Figure S3. Distribution of PI values recorded by FMDV/A-specific mAb-bELISA. Normal distribution of PI obtained from naïve negative sera of Canadian porcine (A), bovine (B), ovine (C), and caprine (D). The red vertical dashed line and grey vertical solid line represent estimated PI cut-off and mean values, respectively.

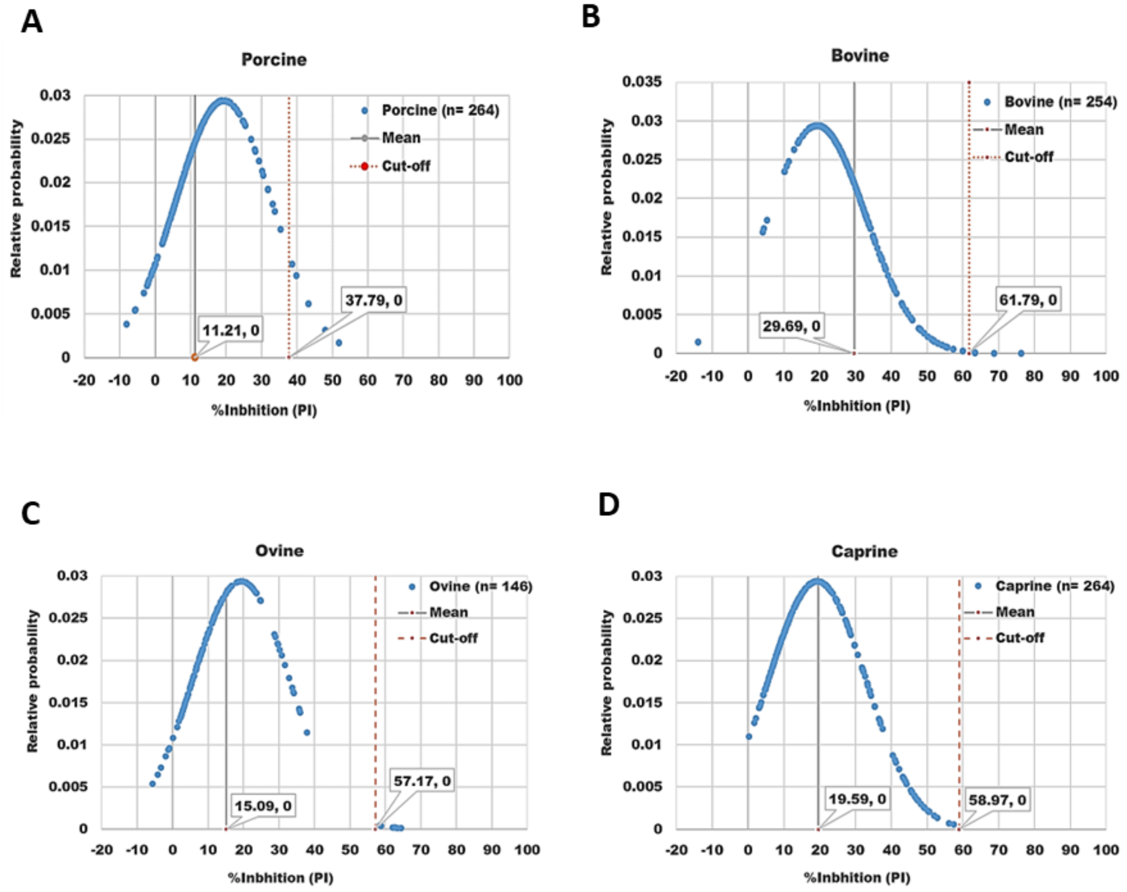

Figure S4. Distribution of PI values recorded by FMDV/Asia1-specific mAb-bELISA. Normal distribution of PI obtained from naïve negative sera of Canadian porcine (A), bovine (B), ovine (C), and caprine (D). The red vertical dashed line and grey vertical solid line represent estimated PI cut-off and mean values, respectively.
